# Music-Enhanced Emotion Identification of Facial Emotions in Autistic Spectrum Disorder Children: A Pilot EEG Study

**DOI:** 10.3390/brainsci12060704

**Published:** 2022-05-30

**Authors:** Rafael Ramirez-Melendez, Elisabet Matamoros, Davinia Hernandez, Julia Mirabel, Elisabet Sanchez, Nuria Escude

**Affiliations:** 1 Music and Machine Learning Lab, DTIC, Universitat Pompeu Fabra, 08018 Barcelona, Spain; 2 Department of Information and Communication Technologies, Universitat Pompeu Fabra, 08018 Barcelona, Spain; elisabet.mlle@gmail.com (E.M.); davinia.hernandez@upf.edu (D.H.); 3 Centre Carrilet, 08031 Barcelona, Spain; juliamiralbell@gmail.com (J.M.); esanchez@carrilet.org (E.S.); 4 Institut Catalá de Musicoterapia, 08021 Barcelona, Spain; nuriescude@ub.edu

**Keywords:** Autistic Spectrum Disorder (ASD), emotions, affective facial expressions, music, brain activity, EEG

## Abstract

The Autistic Spectrum Disorder (ASD) is characterized by a difficulty in expressing and interpreting others’ emotions. In particular, people with ASD have difficulties when interpreting emotions encoded in facial expressions. In the past, music interventions have been shown to improve autistic individuals’ emotional and social skills. The present study describes a pilot study to explore the usefulness of music as a tool for improving autistic children’s emotion recognition in facial expressions. Twenty-five children (mean age = 8.8 y, SD = 1.24) with high-functioning ASD and normal hearing participated in the study consisting of four weekly sessions of 15 min each. Twenty-five participants were randomly divided into an experimental group (N = 14) and a control group (N = 11). During each session, participants in the experimental group were exposed to images of facial expressions for four emotions (happy, sad, angry, and fear). Images were shown in three conditions, with the second condition consisting of music of congruent emotion with the shown images. Participants in the control group were shown only images in all three conditions. For six participants in each group, EEG data were acquired during the sessions, and instantaneous emotional responses (arousal and valence values) were extracted from the EEG data. Inter- and intra-session emotion identification improvement was measured in terms of verbal response accuracy, and EEG response differences were analyzed. A comparison of the verbal responses of the experimental group pre- and post-intervention showed a significant (p = 0.001) average improvement in emotion identification accuracy responses of 26% (SD = 3.4). Furthermore, emotional responses of the experimental group at the end of the study showed a higher correlation with the emotional stimuli being presented, compared with their emotional responses at the beginning of the study. No similar verbal responses improvement or EEG-stimuli correlation was found in the control group. These results seem to indicate that music can be used to improve both emotion identification in facial expressions and emotion induction through facial stimuli in children with high-functioning ASD.

## 1. Introduction

Individuals with Autistic Spectrum Disorder (ASD), including high-functioning ASD, often suffer from difficulties in identifying and sympathizing with mental states, such as emotions or intentions, in others [1]. Thus, ASD is often characterised by difficulties in social and interpersonal communication [2]. Previous studies have shown that it is difficult for people with ASD to identify emotions represented in facial expressions [3,4,5,6,7], in affective speech [7,8,9,10], in non-verbal vocal expressions [11,12], and in body movements [7,13,14]. The difficulties for emotion processing in ASD individuals are associated with abnormal brain activity when compared with neurotypical people, e.g., ASD individuals show less fusiform gyrus and amygdala activity when viewing facial expressions with emotional content [15,16,17,18] and unusual superior temporal and inferior frontal gyrus activation when listening to speech [19,20,21,22].

People with ASD often enjoy music listening, are affected emotionally by music, and are usually musically talented [23,24,25,26]. Previous studies have shown that individuals with ASD process melodic information (i.e., contour and intervals) in the same way as neurotypical people [27], and that they are better at pitch processing [28,29,30] and show superior pitch memory [29,31]. Notably, studies have also shown that individuals with ASD are able to correctly identify emotions in music just as well as neurotypical individuals [23,24,32,33,34]. Previous studies have found that ASD individuals listened to music as often as people without ASD because they feel emotionally affected by it [24]. Furthermore, it has been shown that the physiological responses to music in ASD individuals are the same as for neurotypical people [35], and previous work has observed preserved neural activity for music processing in children with ASD [36]. ASD individuals recruit brain regions involved in the processing of emotion and reward when they listen to happy and sad music, in the same way as neurotypical people do [32].

Music therapy (MT) has been proved to be effective for treating some medical and emotional conditions by using music, e.g., melodies, rhythm, and movement [37,38,39]. Therapists have attempted to take advantage of the musical sensitivity and abilities of ASD individuals to compensate for the social interaction deficits [40,41]. Despite MT being widely used for treating neurological and cognitive disorders [42,43], its application and evaluation for improving social skills in ASD [44,45] remains an open research area. Most of the research on using music as an intervention for ASD has been centred around communication behaviours [46,47]. For instance, Ref. [48] proposed a music intervention based on auditory–motor mapping to improve language development in ASD children with no speech.

There have been several approaches to investigate the influence of music with affective content on individuals with ASD’s ability to identify emotions depicted in visual stimuli. Ref. [49] asked 30 neurotypical children and 20 children with high-functioning ASD to rate expressions (using a seven-point, very sad–very happy) scale of happy, neutral, and sad facial photographs while listening to sad music and happy music. Ratings of happy and neutral faces were unaffected by music conditions, but sad faces were perceived to be sadder with sad music than with happy music. Neurotypical children rated the happy faces as happier and the sad faces as sadder than did participants with ASD. Ref. [50] aimed to investigate the effect of music therapy interventions on teaching the facial expression of sadness to children with ASD. However, the study’s main limitation was that it was conducted with only one participant. Ref. [51] conducted a systematic review of studies investigating the challenges of facial emotion recognition in ASD using eye tracking or EEG. The review indicated a divergence of visual processing pathways in individuals with ASD reflected in observable differences in eye tracking and EEG patterns. Ref. [52] conducted a qualitative study testing the musical empathic ability in participants with an autism spectrum disorder. Their results suggest that people with ASD are able to mirror structural and affective features of the music, concluding that they have an understanding of the affective features of music. Ref. [53] examined the effect of background music and song texts to teach emotional understanding of happiness, sadness, anger, and fear to children with autism. Results showed that participants improved significantly in their understanding of the four selected emotions, with background music significantly more effective than other conditions.

The present study describes a pilot study to explore the usefulness of music as a tool for improving high-functioning ASD children’s emotion recognition in facial expressions. Using facial expressions for four emotions (happy, sad, angry, and fear) with and without congruent affective background music, inter- and intra-session emotion identification improvement was measured in terms of verbal response accuracy and brain activity responses (quantified as arousal and valence levels).

## 2. Materials and Methods

### 2.1. Participants

Participants included in this study were twenty-five children aged 6–11 years (all male, M = 8.8 y, SD = 1.2) with high-functioning autistic spectrum disorder (ASD) attending C.E.E. Carrilet and the Music Therapy Catalan Institute, Barcelona. The diagnosis of ASD was performed by an experienced clinician on the basis of DSM-V criteria, the children’s current situation, and developmental history. Diagnoses were confirmed using the Autism Diagnostic Observation Schedule [54]. Global intelligence was measured using either the Wechsler Intelligence Scale for Children–Fourth Edition [55] or the Wechsler Non-Verbal Scale of ability (WNV) [56] depending on children’s verbal abilities (IQ ≥ 80). Children with psychiatric disorders were not considered. Written informed consent was obtained from the parents of the participants, and the study procedures were positively evaluated by the Clinical Research Ethical Committee of the Fundació Unio Catalana Hospitals, Barcelona, Spain, under reference number CEIC 15/55.

### 2.2. Materials

#### 2.2.1. Facial Expression Database

The images employed in the study were drawn from the Karolinska Directed Emotional Faces database (1998) created at the Department of Clinical Neuroscience, Karolinska Institutet, Stockholm, Sweden. These pictures are intended as a tool for medical and psychological purposes related to perception, emotion, and memory. Facial expressions were taken from 5 different angles with a uniform light and position of participants’ eyes and mouths. Participants were 70 volunteers equally clothed, 35 females and 35 males, ages ranging from 20–30 years. They displayed a total of 7 different emotions (i.e., disgust, happiness, sadness, fear, anger, neutral, and surprise), which resulted in a set of 4900 pictures of 562 × 762 pixels. In this study, a total of 4 emotions were selected: fear, happiness, sadness, and anger. We used a total of 36 different pictures per session (i.e., 12 images per condition, 6 males and 6 females); each emotion was displayed by a different person, and no picture was selected more than once. Those pictures considered unclear were discarded.

#### 2.2.2. Music Material

Music used in the study was drawn from the dataset of a soundtrack for music and emotion created at the University of Jyväskylä, Finland. The dataset consists of a set of 360 audio clips of soundtracks that had been specifically composed to trigger 5 emotional states: anger, fear, relaxation, happiness, and sadness. Excerpts were 20 s in duration each; did not contain lyrics, dialogue, or sounds effects (e.g., car sounds); and were not familiar to any of the participants. In the study we used audio clips for anger, fear, happiness, and sadness in order to match the emotions portrayed by the selected visual stimuli.

#### 2.2.3. Data Acquisition and Processing

**Figure 1:**
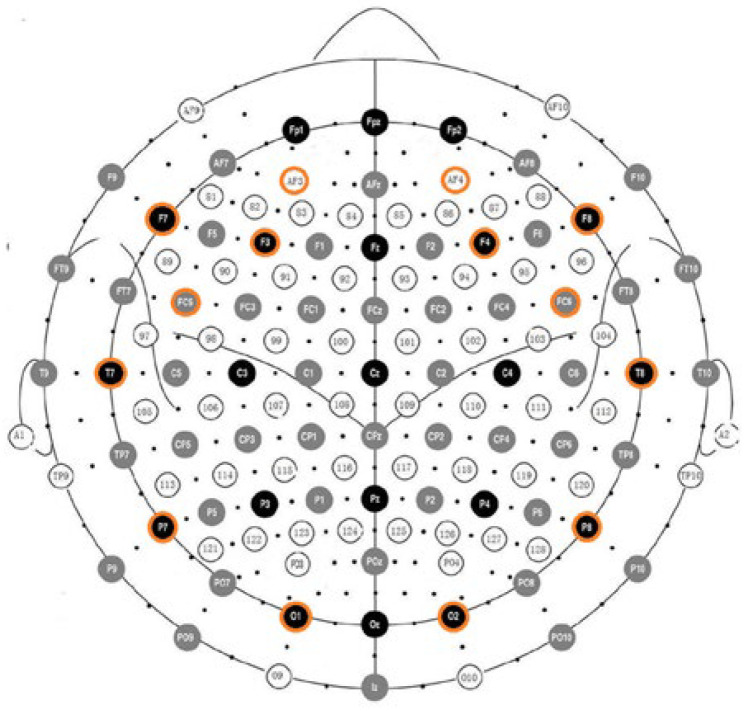
International 10–20 system showing the electrode positions in the Emotiv EPOC.

EEG data were acquired using the Emotiv EPOC EEG system (Emotiv, San Francisco, CA, USA, 2014). The system consists of 16 wet saline electrodes, 14 EEG channels, and a wireless amplifier. Electrodes were located at AF3, F7, F3, FC5, T7, P7, O1, O2, P8, T8, FC6, F4, F8, and AF4 according to the international 10–20 system (see Figure 1). Reference electrodes were located at P3 and P4 (above the participants’ ears). Data were digitized using the Emotiv EPOC built-in 16-bit ADC with a 128 Hz sampling frequency per channel and sent to the computer via Bluetooth. The resulting EEG data were filtered using Butterworth 8–12 Hz and 12–28 Hz filters. The Emotiv Control Panel software (version 1.0.0.5, San Francisco, CA, USA) was used to visually monitor electrode contact impedance to the scalp.

The Emotiv EPOC EEG device is a low-cost EEG device that captures a lower quality signal compared to other more expensive equipment. However, low-cost EEG devices can be reliable for measuring EEG signals for research purposes [57,58,59]. A review of the Emotiv EPOC EEG device, as well as of other low-cost systems, can be found in [57]. For recording and processing the EEG data, as well as for synchronously presenting the images and audio clips, the OpenViBE platform [60] was used.

### 2.3. Methods

#### 2.3.1. Experimental Design

We conducted a controlled study through the course of 4 consecutive weeks. Using the method of randomly permuted blocks, participants were randomly assigned into two groups: an experimental group EG (N = 14) and a control group CG (N = 11). Each participant was exposed to three sequential conditions in each session. Conditions for the EG participants were no-music (NM1), music (M), and no-music (NM3), while participants in the CG were exposed to 3 no-music conditions (NM1, NM2 and NM3). In each condition, a total of 12 stimuli with emotional content (3 happy, 3 sad, 3 angry, and 3 fear) were presented in random order to the participants. The stimuli presented in conditions NM1, NM2, and NM3 were images drawn from the Karolinska Directed Emotional Faces database one at a time, while in condition M, in addition to the facial expression image, an emotion-matching music excerpt from the soundtrack database was concurrently presented. In each condition, 12 stimuli were presented in random order (6 males + 6 females). No image nor music excerpt was presented twice to a participant during the study. The stimuli duration was 10 s with a 5 s transition (i.e., a black screen and no sound) among stimuli. During stimuli transitions, participants responded to the question, “how is this person feeling?” No instructions about their replies to the question were given to participants. Participants’ verbal and EEG activity responses were recorded. The person collecting the responses stood behind the participants, so participants could not see or receive any facial, vocal, or bodily gesture cues from her. Participants did not receive any type of feedback about the correctness of their replies during the whole study. No incentives were given to the children participating in the study, and all of them were willing to participate following the instructions.

#### 2.3.2. Statistical Analysis

Verbal responses were analysed using the SPSS statistics software (IBM Corp., New York, NY, USA, 2010) [61]. We were interested in testing two hypotheses regarding verbal responses: (1) if there was an improvement in responses accuracy within the same session between the first and the third conditions and (2) if there was an improvement in responses accuracy in the last session compared to the first session for the first condition. For testing the first hypothesis, the assumption for normality was tested through the Shapiro –Wilk test for normality (*p* ≤ 0.05), resulting in data that differed significantly from a normal distribution. A Wilcoxon matched-pairs signed-ranks test was performed to check whether there had been an improvement within the same session between the first and the third conditions. In order to test the second hypothesis, the normality of the data in the first condition for all sessions was tested with the Shapiro–Wilk test for normality (*p* ≥ 0.05); data did not differ from a normally distributed data set. A test for a within-subjects, repeated-measures study design was performed so as to verify whether there was a significant difference through the course of the sessions. A paired-samples ANOVA was run, thus allowing us to contrast the scores in the first condition along the intervention.

#### 2.3.3. EEG Analysis

**Figure 2:**
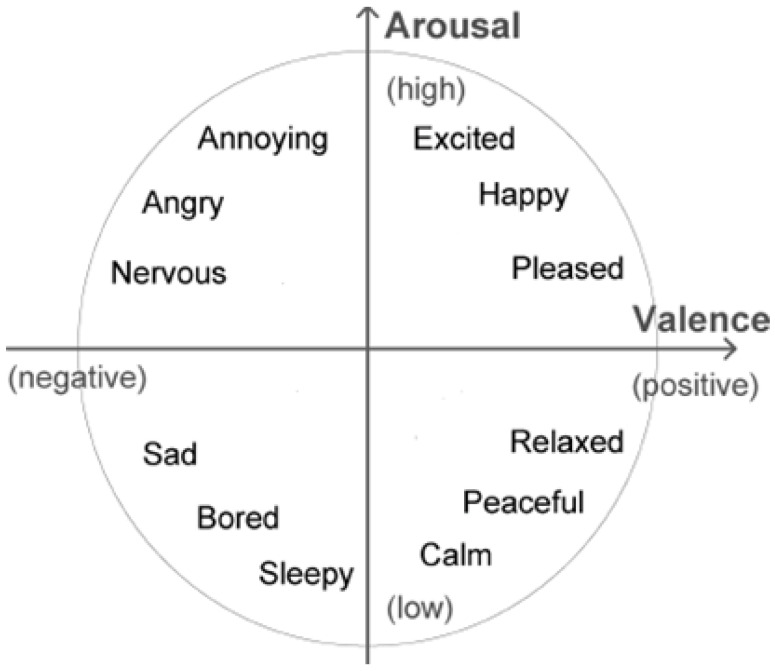
Thayer’s arousal–valence emotional plane.

EEG data recorded from participants was normalized and transformed into arousal and valence values in the Thayer’s emotion plane [62], depicted in Figure 2. EEG data were processed following Ramirez et al. [63,64]. In Ramirez et al. (2012), it was shown that the computed arousal and valence values contain meaningful information about the user’s emotional state. Artifact detection/elimination was performed by a visual inspection of the signal. Arousal levels were computed as the ratio of EEG beta (12–28 Hz) and alpha (8–12 Hz) brainwaves recorded at 4 locations on the prefrontal cortex: AF3, AF4, F3, and F4 (see Figure 1). Concretely, instantaneous arousal levels were computed as specified by Equation (1):

Arousal = (βF3 + βF4 + βAF3 + βAF4)/(αF3 + αF4 + αAF3 + αAF4)(1)

Motivated by previous EEG studies [65,66,67,68], valence values were computed as the difference between alpha power α in the right and left frontal area (i.e., in channels F4 and F3). More precisely, valence levels were computed as specified by Equation (2):

Valence = αF4 − αF3(2)

The above arousal level computation is motivated by the fact that beta waves are associated with alert and excited states of mind, whereas alpha waves are more dominant in a relaxed state. Thus, the beta/alpha ratio is a reasonable indicator of the arousal state of a person. Similarly, valence computation is motivated by psychophysiological research, which has shown the importance of the difference in activation between the cortical hemispheres. Left frontal inactivation is an indicator of a withdrawal response, which is often linked to a negative emotion. On the other hand, right frontal inactivation may be associated to an approach response, or positive emotion. Positions AF3, F3, AF4, and F4 are the most commonly used positions for computing arousal and valence, as they are located in the prefrontal lobe, which plays a central role in emotion regulation. More details about the way arousal and valence are computed can be found in [64].

For each condition, EEG data were segmented according to the different emotional stimuli presented, i.e., EEG data were divided into data recorded during the presentation of happy, sad, angry, and fear emotion stimuli. For the non-music (NM) condition in the first session and post session four, machine learning techniques were applied to train computational models to predict the class of stimuli (happy, sad, angry, and fear) from the arousal/valence descriptors extracted from the EEG activity. Concretely, the EEG signal was processed to extract instantaneous arousal and valence values, and these values were used to train an artificial neural network (2-node input layer, two 3-node hidden layers, and 4-node output layer) with happy, sad, angry, and fear as target classes. The predictive model was evaluated using stratified 10-fold cross validation. In addition, for each class and all participants, arousal–valence centroids for the NM1 condition of session one and post session four were computed.

## 3. Results

A matched pairs signed-ranks test was performed to check whether there was an improvement in the verbal responses accuracy within the same session between the first (NM1) and the third (NM3) conditions. For both the EG and the CG, the test indicated that there was no statistically significant difference between NM1 and NM3, failing to confirm that in the EG the music stimuli condition (M) had an immediate residual effect on the NM3 condition within sessions.

A test for a within-subjects repeated measures study design was performed so as to verify whether there was a significant difference amongst the verbal responses accuracy of the first and last session. With this purpose, we compared the verbal responses accuracies in the NM1 condition of the first session with an NM1 a week after the last session. Table 1 shows the statistics of verbal responses of condition NM1 (first session) and condition NM1 a week after the last session for both the EG and the CG. The results show a statistically significant effect in the EG (*p* = 0.011), while no significance was found in the CG (*p* = 0.695). This result shows that the EG participants’ scores in NM1 had significantly increased at the end of the study with respect at to the beginning of the study. It is important to note that participants did not receive any type of feedback about the correctness of their replies during the whole study. The person collecting the responses was, at all times, standing behind the participants, so participants could not see or receive any facial, vocal, or bodily gesture cues from the experimenter. This rules out the possibility that the resulting significant improvement in accuracy was simply due to habituation or practice. Thus, such improvement seemed to be due to the effect of music.

**Figure 3:**
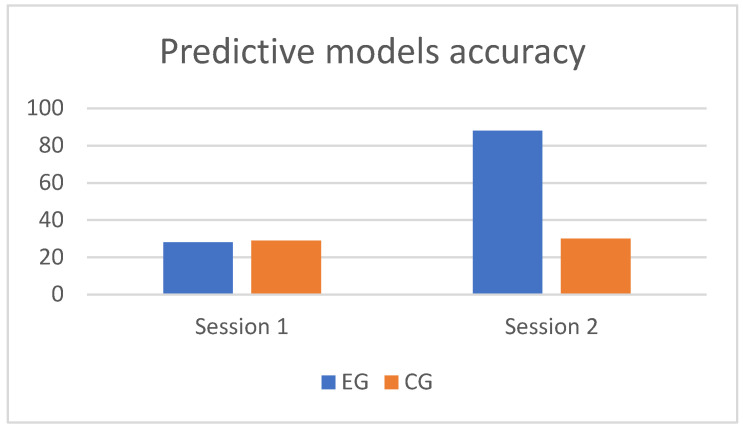
Correctly Classified Instances percentages (C.C.I.%) of classification models obtained by training with the arousal and valence values for NM1 session 1 and NM1 post session 4.

The accuracies of the computational predictive models obtained by training an artificial neural network with the arousal and valence values for condition NM1 in session one and condition NM1 a week after the last session for participants in the EG and CG are shown in Figure 3. The difference between NM1 session one and NM1 a week after the last session in the EG was significant (*p* = 0.002), while no significant difference was found in the CG.

**Figure 4:**
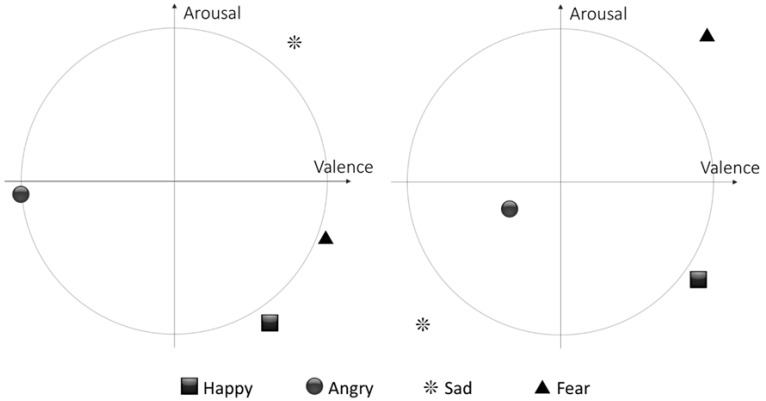
Session 1 and post session 4 normalized averaged (centroids) arousal and valence values in NM1 condition for the four emotional stimuli considered.

Average arousal and valence values were computed for each type of emotion stimuli in condition NM in session one and in session four. Figure 4 shows normalized averages plotted in the arousal (y-axis) valence (x-axis) plane.

## 4. Discussion

No significant difference in verbal responses accuracy between the two non-music conditions (NM1 and NM2) in the same session was found. This confirms that the music stimuli in the intermediate condition (M) had no immediate residual effect on NM2 in terms of verbal responses. This result is not surprising since it seems unreasonable to expect a significant effect of a sole music condition. However, the effect of two consecutive sessions on a third one (S1–S3 and S2–S4) was found significant. As expected, the global effect of the study (S1–S4) was the most significant. Interestingly, the effect of the second session (S2) on the third one (S3) was also found to be significant. The reason for this may be that S1 had an accumulative effect on S2 and thus on S3.

The difference between the classification accuracy obtained by the trained models and the accuracy of the baseline classifier (25% in the case of the balanced four-class emotion classification task) indicates that arousal and valence indicators extracted from the EEG data contain sufficient information to distinguish the emotional states produced by the different stimuli, and that the machine learning method applied is capable of learning the EEG patterns that distinguish these states. It is worth noting that the machine learning algorithm investigated, i.e., artificial neural networks (ANN), produced significantly better results than random classification accuracies for every participant. This supports our idea about the feasibility of training classifiers for the cognitive states produced by the different emotional stimuli considered. It is also worth noticing that ANN produce non-linear models, so we tested support vector machines (SVM) with a linear kernel to see if the results were maintained. The results of the SVM model were not significantly better than the baseline classifier for most of the participants, suggesting that the data are non-linearly distributed in the arousal–valence plane.

The obtained models’ classification accuracies may be interpreted as an indicator of how differentiable the participants’ emotional responses (estimated by their computed arousal and valence responses) are during different stimuli presentation. This is, if it was the case that stimuli did not have any emotional effect whatsoever in the participants and thus did not produce any differentiable EEG responses, the accuracies of the models would be expected to be close to baseline (25%). The fact that the participants’ emotional responses are consistently more easily classified post fourth session compared with the first session may indicate that the repeated presentation of the stimuli presentation throughout the study has an effect on the neural encoding of the emotional stimuli. However, this does not imply that the resulting neural encoding does actually correspond with the emotion being presented. In order to investigate the neural encoding relation with the stimuli, presented arousal and valence indicators were computed at the beginning and the end of the study.

Average arousal and valence values were computed for each type of emotion stimuli in session one and post session four for condition NM. Figure 4 (left) shows the result for session one: there is no correlation between the relative arousal and valence values and the emotion presented in the corresponding stimuli. In Figure 4 (right), which shows the result for post session four, there is a partial correlation between the relative arousal and valence values and the emotion presented in the corresponding stimuli. Happy, sad, and angry stimuli seem to be encoded as expected according to the EEG activity, although the valence of fear stimuli appears to be wrongly encoded.

This pilot study has several limitations. First, in order to be able to draw more definite conclusions about the benefits of using music as a tool for improving facial emotion recognition in children with ASD, ideally a neurotypical children group exposed to visual and auditory stimuli should be included. In this way it would have been possible to quantify both the added value of music for improving facial emotion recognition in children with ASD and comparing the results of the ASD study with the results of neurotypical children. A second limitation of the presented study is the reduced number of participants. Unfortunately, we were not able to have access to a larger sample of children with ASD. Given these limitations, the present pilot study should be extended taking into account these issues. Another possible limitation of the current study is the reduced quality signal of the Emotiv EPOC device. However, in the case of this study, the Emotiv EPOC provided important pragmatic advantages when compared with more expensive equipment: The Emotiv EPOC system’s setting-up time is considerably shorter than that of an expensive EEG system. Furthermore, expensive EEG systems typically require the application of conductive gel for a reliable electrode connection, which can be inconvenient (i.e., the gel attaches to the hair, which requires the entire head to be washed at the end of each session). In the case of the Emotiv EPOC, the setting-up time takes a few minutes (typically 3–5 min), and conductive gel is not necessary. In the case of (ASD) children participants, these are important issues to be considered.

In summary, the use of music seemed to produce a significant improvement in the emotion identification accuracy of verbal responses within four sessions. Furthermore, participants’ emotional responses computed from their EEG data post the last session showed a better correlation with the emotional stimuli being presented, compared with their emotional responses in the first session. Results seem to indicate that music can be used to improve both emotion identification and emotion induction of facial expressions in children with high-functioning ASD.

## Figures and Tables

**Table 1:** Statistics of verbal responses of condition NM1 (S1) and NM1 (post S4).

Group	Mean	SD	t-Value	*p*-Value
EG	1.667	0.422	3.953	0.011
CG	0.167	0.983	0.415	0.695

## Data Availability

Upon request.
